# Hospital Sunday in London

**Published:** 1910-06-11

**Authors:** 


### The Hospital

A Journal of

The Medical Sciences and Hospital Administration.

New Series. No. 174, Vol. VII. [No. 1246, Vol. XLVIII.] SATURDAY, JUNE 11, 1910

### Hospital Sunday in London.

Next Sunday is Hospital Sunday in London, and if it does not result in a considerable increase in the individual and total collections from churches and places of worship, only one conclusion will be possible. During the last six years, with the exception of a slight recovery in 1905, the amounts received from congregations have shown a continuous diminution. In 1903 the congregations collected upwards of £49,000; in 1909 they only contributed a little over £39,000, a drop of over 20 per cent. The lessened interest in these collections on the part of all concerned is emphasised by the circumstance that, whereas only 1,987 congregations contributed in 1903, 2,070 congregations made collections in 1909. Whether the fault rests with the clergy and ministers of religion, with the people, the Fund organisation, or is due to diminishing congregations and the gradual falling off in the number of worshippers and the influence of religious teachers, are questions which should, we think, be faced and determined at the proper time by a special committee appointed with full power to go into these questions and ascertain the facts. We hope, however, that such a step may prove unnecessary for the reasons pointed out in the special Hospital Sunday number of The Hospital, which deals with King Edward VII.'s life-work for the hospitals. It is incredible, with the example of King Edward before them, that every clergyman and minister of religion in London on Sunday next will not make a special and strenuous effort to secure the maximum sum ever raised from the congregations as a living memorial to our lamented King's memory. They have a great opportunity as well as a plain duty and privilege, which are to set an example and to drive home to every man, woman, and child in the metropolis the works of mercy which King Edward VII. loved to further, and which constitute the special feature of Hospital Sunday.

Of course, the magnificent bequest of the late George Herring to the Hospital Sunday Fund, which has yielded an average of over £30,000 a year in revenue during the last three years, may have had an adverse effect upon the collections. If this be true it can only have arisen from misapprehension, for the voluntary hospitals of London need a contribution of £100,000 a year through Hospital Sunday, and it has been the aim and endeavour of the most active organisers of the Sunday Fund to secure that amount. Mr. George Herring had great sympathy with this movement, and hoped by his bequest to encourage the congregations to raise their annual gifts sufficiently to provide £100,000 per annum for the hospitals.

Preachers would do well to make a point of calling attention to the reasons which influenced Mr. Herring, no doubt, to give so large a sum of money to the metropolitan hospitals through the Hospital Sunday Fund. During his life he added twenty-five per cent., or five shillings in the pound, and sometimes more, to the amount of each congregational collection, with the object of raising the total to an adequate sum. This action on Mr. Herring's part proves to demonstration what his motive was. We are confident that if the facts are brought home, as they ought to be, by each preacher to the hearers at every service held in London next Sunday, all who have been genuinely moved by the sudden death of our beloved King will quietly give expression to the ocean of sympathy which has been aroused. If they do this, their gifts will take a form which our late King would have particularly appreciated.

We feel it a public duty to point out these facts, to endeavour to focus public opinion and to move every man who may be called upon to preach on June 12, 1910, to do his utmost to lift the Hospital Sunday Fund out of the relatively unsatisfactory condition into which it has fallen, from circumstances which the present occasion should change for the better, by placing the Fund upon the financial basis which Mr. George Herring and others have striven for years to secure.

If Hospital Sunday in London this year cannot be made to yield, through the congregations alone, a record sum, we say with some confidence that the failure to secure such a result, which in the circumstances should be readily attainable, must be accepted by those who have the best interests of the Fund most closely at heart, as the strongest evidence that there is an urgent necessity to take the Fund seriously in hand, and to place its organisation upon a more modern and fruitful basis.

